# Population structure of *Clinostomum complanatum* (Trematoda: Digenea) with new data on haplotype diversity of flukes from Slovakia and Italy

**DOI:** 10.1051/parasite/2024080

**Published:** 2025-01-22

**Authors:** Ľudmila Juhásová, Eva Čisovská Bazsalovicsová, Monica Caffara, Alžbeta Radačovská, Andrea Gustinelli, Lucia Dinisová, Yaroslav Syrota, Ivica Králová-Hromadová

**Affiliations:** 1 Institute of Parasitology, Slovak Academy of Sciences Hlinkova 3 040 01 Košice Slovakia; 2 Department of Veterinary Medical Sciences, Alma Mater Studiorum University of Bologna Via Tolara di Sopra 50 40064 Ozzano Emilia, Bologna Italy; 3 The University of Veterinary Medicine and Pharmacy in Košice Komenského 73 041 81 Košice Slovakia; 4 I. I. Schmalhausen Institute of Zoology of National Academy of Sciences of Ukraine B. Khmelnytskogo 15 01054 Kyiv Ukraine

**Keywords:** Yellow grub, Clinostomiasis, Haplotype network, Mitochondrial DNA, Cytochrome c oxidase subunit 1

## Abstract

The fluke *Clinostomum complanatum*, a parasite of piscivorous birds, but also reptiles and rarely mammals, has established several foci in the western Palaearctic regions. Previous studies pointed out the complicated taxonomy of the genus, but broader population genetic analysis of *C. complanatum* has not yet been carried out. The aim of this study was to determine the structure, intraspecific variability, and diversity of mitochondrial *cox*1 haplotypes of *C. complanatum* from different localities in Slovakia (Danube floodplain forests) and Italy (Emilia-Romagna and Tuscany), as well as to evaluate the interrelationships among populations from Europe, the Middle East, and North Africa. The genetic structure of *C. complanatum* from Slovakia and Italy was represented by a great number of haplotypes, showing stable populations with high intraspecific diversity. The haplotypes of samples from other localities (Romania, Turkey, Egypt, and Iran) showed possible gene flow among the populations from Central Europe down to the Mediterranean region, North Africa, and the Middle East. The genetic homogeneity of these samples can be linked to the distribution and migratory routes of the definitive hosts, aquatic piscivorous birds, mainly herons and cormorants, that spread parasite eggs among the continents.

## Abstract (fr)

La douve *Clinostomum complanatum*, un parasite des oiseaux piscivores, mais aussi des reptiles et rarement des mammifères, a établi plusieurs foyers dans les régions paléarctiques occidentales. Des études antérieures ont souligné la taxonomie compliquée du genre, mais une analyse génétique de la population plus large de *C. complanatum* n’avait pas encore été réalisée. L’objectif de cette étude était de déterminer la structure, la variabilité intraspécifique et la diversité des haplotypes mitochondriaux *cox1* de *C. complanatum* de différentes localités de Slovaquie (forêts de la plaine inondable du Danube) et d’Italie (régions d’Émilie-Romagne et Toscane), ainsi que d’évaluer les interrelations entre les populations d’Europe, du Moyen-Orient et d’Afrique du Nord. La structure génétique de *C. complanatum* de Slovaquie et d’Italie était représentée par un grand nombre d’haplotypes, montrant des populations stables avec une grande diversité intraspécifique. Les haplotypes d’échantillons provenant d’autres localités (Roumanie, Turquie, Égypte et Iran) ont montré un possible flux génétique entre les populations de l’Europe centrale jusqu’à la région méditerranéenne, l’Afrique du Nord et le Moyen-Orient. L’homogénéité génétique de ces échantillons peut être liée à la distribution et aux voies migratoires des hôtes définitifs, des oiseaux aquatiques piscivores, principalement hérons et cormorans, qui propagent les œufs des parasites entre les continents.

## Introduction

The fluke *Clinostomum complanatum* (Rudolphi, 1814) Braun, 1899 is the type species of the genus *Clinostomum* Leidy, 1856 (Digenea: Clinostomidae). The typical multi-host life cycle of this trematode comprises two intermediate hosts: aquatic pulmonate snails (1st intermediate hosts) and freshwater fish or amphibians (2nd intermediate hosts) [2, 22]. Endothermic piscivorous vertebrates, mainly birds, but also reptiles and rarely mammals (including humans), serve as definitive hosts of *C. complanatum*, in which the parasite resides in the oral cavity or oesophagus [13].

**Figure 1:**
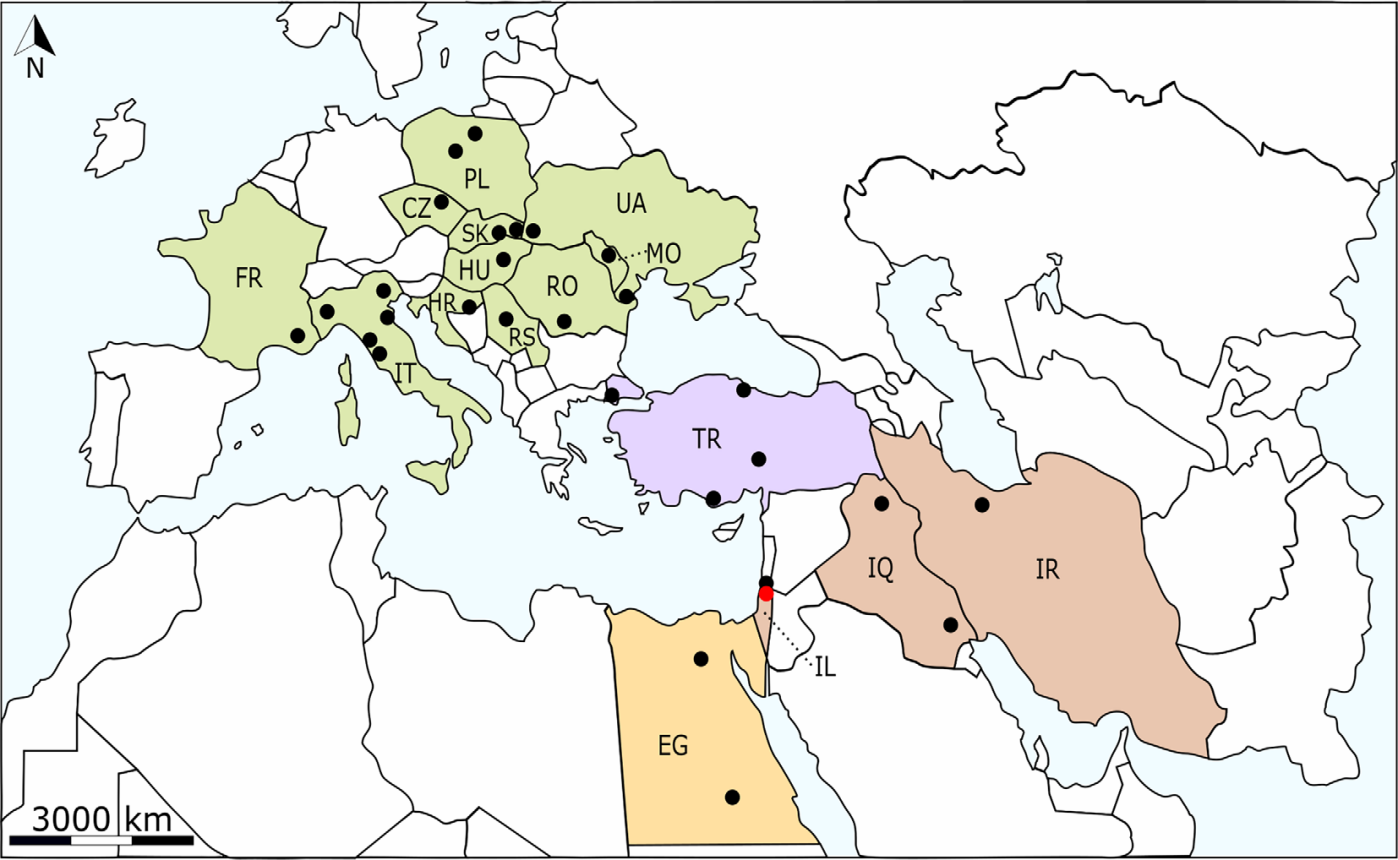
Schematic representation of the distribution of *Clinostomum complanatum* in Europe, the Middle East, and North Africa. Colours of the individual geographical regions: green – Europe: HR, Croatia; CZ, Czechia; FR, France; HU, Hungary; IT, Italy; MO, Moldova; PL, Poland; RO, Romania; RS, Serbia; SK, Slovakia; UA, Ukraine; purple – Europe/Asia: TR, Turkey; orange – North Africa: EG, Egypt; brown – the Middle East: IR, Iran; IQ, Iraq; IL, Israel. Black dots, *C. complanatum* infections in wildlife (fishes and birds); red dot, infection in humans. The basemap was obtained from https://d-maps.com.

The distribution of *C. complanatum* is confined to the western Palaearctic regions, mainly in Europe, but also in the Middle East and North Africa [21] (Fig. 1). The fluke has several foci in Europe, particularly in the southern Mediterranean countries (Italy, France, and Croatia), in the Balkan region (Romania and Serbia), as well as in Eastern (Moldova and Ukraine) and Central Europe (Czechia, Slovakia, Hungary, and Poland) (Fig. 1; Supplementary Table 1).

*Clinostomum complanatum* has been intensively studied in Italy, mainly in the Emilia-Romagna region. Metacercariae were found in multiple families of fish (mainly Cyprinidae, but also Cobitidae and Percidae), and in two amphibian species (Salamandridae), while adult flukes were found in birds of the family Ardeidae. In Slovakia, metacercariae of *C. complanatum* have been determined in Cyprinidae, Cobitidae, and Percidae from eastern Slovakia and the Danube River basin (south-west Slovakia). Adult flukes were found only in the purple heron *Ardea purpurea* from the Danube region. Several findings of *C. complanatum* metacercariae have been reported in fish from Romania, while single findings were documented in Poland, Croatia, Czechia, France, Hungary, Moldova, Serbia, and Ukraine. Supplementary Table 1 provides details of hosts, localities, and relevant references.

The parasite has also been detected in fish from Turkey and in fish and the great cormorant *Phalacrocorax carbo* in Egypt. Another focus of *C. complanatum* is in the Middle East, namely in Iran, Iraq, and Israel. Most of the data originated from Iran, where the flukes have been described mainly in various cyprinids, while there is only one record each from Iraq and Israel. Adults of *C. complanatum* have been found in various Ardeidae species from all three Middle Eastern countries (see Supplementary Table 1 for details and references). The only human infection with *C. complanatum* was documented in Israel 80 years ago by Witenberg [35]. The author identified a worm extracted from the throat of a patient from Tiberias, a city on the western shore of the Sea of Galilee (Tiberias Lake). This human infection was linked to the observed *C. complanatum* infections of fish from the Tiberias Lake.

Recently, Locke *et al*. [21] analysed the complete mitochondrial genomes of *C. complanatum* from Europe (Italy) and Asia (China) and published ground-breaking data suggesting separation of *Clinostomum* species in the western and eastern Palaearctic. The authors described the new species *Clinostomum sinensis* based on morphological comparisons and molecular analyses, and synonymised it with the previously identified *C. complanatum* from Japan [18] and China [3, 20]. Later, Monnens *et al*. [26] analysed the mitogenome of *C. complanatum* from Iran and revealed a high genetic similarity with the mitogenome of *C. complanatum* from Italy [21] and differences from the mitogenome of *C. sinensis* from China [3]. The description of *C. sinensis* showed that the taxonomy of *Clinostomum* from Southeast Asia (South Korea, Japan, Taiwan, India, Thailand, and China) (see Supplementary Table 2 for details and references) requires critical reappraisal.

Locke *et al*. [21] emphasised the need for a comprehensive, multidisciplinary approach for reliable species identification and assessment of the species diversity in the genus. However, all published studies to date have been conducted on local populations of *C. complanatum* and a broader population genetic analysis has not yet been performed. Therefore, the objective of the present work was to determine the genetic structure, intraspecific variability, and haplotype diversity of the mitochondrial cytochrome *c* oxidase subunit 1 (*cox*1) of newly studied populations of *C. complanatum* from different localities in Slovakia and Italy, as well as to evaluate the interrelationships among populations from Europe, the Middle East, and North Africa.

## Materials and methods

### Ethics

The fish were caught by professional fishermen under permit no. 12-1-2024 issued by the Ministry of the Environment of Slovakia, permission no. 29234/2012 issued by the Italian Institute for Environmental Protection and Research (ISPRA), and permission no. 1120 2008 7123 1588 9462 issued by the Regione Emilia-Romagna (12781 – RERLIP). All methods used in this study were carried out in accordance with the relevant guidelines and regulations (Decree of the Ministry of the Environment of Slovakia No. 381/2018 Coll. and Act No. 216/2018 Coll. about fishing).

### Parasitic material

A total of 157 *C. complanatum* metacercariae from Slovakia and Italy were analysed in the current work. In total, 55 flukes from Slovakia were isolated from the musculature of the European perch *Perca fluviatilis*. The sampling area was located in the central region of the Danube River in south-west Slovakia. Four river arms (RAs) were located directly on the main stream of the Danube, namely Starohájske RA (*n* = 5), Karloveské RA (*n* = 4), Jarovecké RA (*n* = 11), and Biskupické RA (*n* = 24). Šulianske Lake (*n* = 11), a gravel pit permanently flooded with water, is located outside but close to the Danube River.

**Figure 2:**
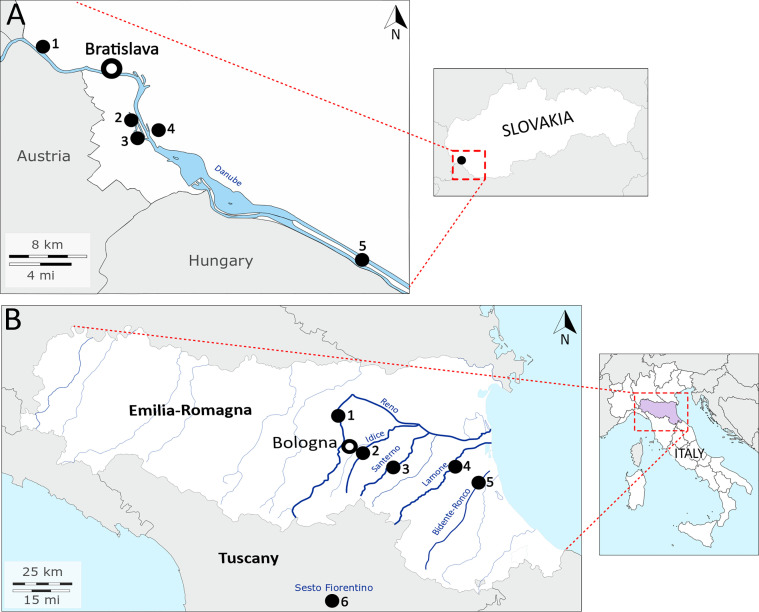
Details on sampling sites of *Clinostomum complanatum* from Slovakia (A) and Italy (B). (A) Danube River in south-west Slovakia; 1, Karloveské river arm; 2, Starohájske river arm; 3, Jarovecké river arm; 4, Biskupické river arm; 5, Šulianske Lake. (B) Emilia-Romagna region and Tuscany in Italy; 1, Reno River, Case Reno Sabbioni; 2, Idice River, Borgatella; 3, Santerno River, Codrignano; 4, Lamone River, Brisighella; 5, Bidente-Ronco River, Coccolia; 6, Sesto Fiorentino. The basemaps were obtained from https://d-maps.com.

For Italy, 102 metacercariae were included in the study. Two samples were collected from the amphibian smooth newt *Lissotriton vulgaris* from an artificial pond near Sesto Fiorentino in the Tuscany region, central Italy. A total of 100 metacercariae were isolated from fish (European chub *Squalius cephalus* and barbel *Barbus barbus*) from the rivers Reno (*n* = 20), Idice (*n* = 38), Santerno (*n* = 11), Lamone (*n* = 29), and Bidente-Ronco (*n* = 2) located in the Emilia-Romagna region, northern Italy. Table 1 summarises the localities and hosts of analysed material and Figure 2 shows the schematic geographical position of studied localities.

**Table 1:** Localities, hosts, and numbers of *Clinostomum complanatum* analysed in the current work.

Country	Locality	Sample code	Coordinates	Host	No.
Slovakia (SK)	Starohájske river arm (ST)	SK-ST	48.1031 N, 17.1322 E	*Perca fluviatilis*	5
	Karloveské river arm (KA)	SK-KA	48.1461 N, 17.0639 E	*P. fluviatilis*	4
	Jarovecké river arm (JA)	SK-JA	48.0756 N, 17.1399 E	*P. fluviatilis*	11
	Biskupické river arm (BI)	SK-BI	48.0876 N, 17.1622 E	*P. fluviatilis*	24
	Šulianske Lake (SL)	SK-SL	47.9407 N, 17.4284 E	*P. fluviatilis*	11
				**Subtotal**	**55**
Italy (IT)	Idice River, Borgatella (ID)	IT-ID	44.4385 N, 11.4413 E	*Barbus barbus*	38
	Santerno River, Codrignano (SA)	IT-SA	44.3386 N, 11.6991 E	*B. barbus*	11
	Bidente-Ronco River, Coccolia (BI)	IT-BI	44.3041 N, 12.1097 E	*Squalius cephalus*	2
	Reno River, Case Reno Sabbioni (RE)	IT-RE	44.7372 N, 11.5101 E	*B. barbus*	20
	Lamone River, Brisighella (LA)	IT-LA	44.1338 N, 11.4722 E	*S. cephalus*	29
	Sesto Fiorentino (SF)	IT-SF	43.8247 N, 11.1732 E	*Lissotriton vulgaris*	2
				**Subtotal**	**102**
				**Total**	**157**

### Comparative GenBank data on *cox*1 of *C. complanatum*

The objective of this work was to assess the genetic structure and haplotype diversity of *cox*1 mtDNA of *C. complanatum* from Slovakia and Italy, and to evaluate the genetic relationships among populations from Europe, the Middle East, and North Africa. To achieve these aims, >200 GenBank records (https://www.ncbi.nlm.nih.gov) were analysed in detail to summarise all complete and partial *cox*1 sequences of *C. complanatum* available in this database and/or published in the literature. The aim of the analysis was: (1) to obtain the comparative *cox*1 sequence data for *C. complanatum* from different localities for a population study, and (2) to design *C. complanatum*-specific primers for amplification of the *cox*1 region in newly analysed flukes from Italy and Slovakia.

*Clinostomum complanatum* has been detected in 11 European countries (Fig. 1; Supplementary Table 1), but *cox*1 sequences were only available for flukes from Italy and Romania (Table 2). A complete *cox*1 sequence (1,557 bp) identified within the complete mitochondrial genome (13,727 bp) was available for one specimen from Italy [21], while partial *cox*1 sequences (345–622 bp) were obtained for 19 specimens from Italy [1, 2, 13] and two from Romania [21]. Another complete *C. complanatum cox*1 sequence (1,557 bp), identified within the complete mitochondrial genome (14,395 bp), was recently reported from Iran by Monnens *et al*. [26]; five partial sequences were available from Iran [26], one from Iraq, eight from Turkey [32], and four from Egypt [31] (see Table 2 for details and accession numbers).

**Table 2:** Summary of the complete and partial sequences of the mitochondrial *cox*1 gene of *Clinostomum complanatum* from GenBank.

Country (code)	Locality	Host species (Type)	Acc. number	No.	Sequence size (bp)	References
**Complete sequences**						
Italy (IT)	Santerno River	*Squalius cephalus* (F)	MK814187	1	1,557	[21]
Iran (IR)	Mazandaran Province	*Nycticorax nycticorax* (B)	OP681143	1	1,557	[26]
**Partial sequences**						
Turkey (TR)	Central Anatolia Region	*S. cephalus* (F)	MF928768–MF928774	7	582	[32]
	Samsun	*Ardea cinerea* (B)	MT602068	1	557	Unpublished
Iran (IR)	Northwestern Iran	*Cyprinus carpio* (F)	OP709260–OP709261	2	350	Unpublished
	Aras River	*C. carpio* (F)	OP984764	1	620	Unpublished
	Isfahan City	*Capoeta capoeta* (F)	OP678025–OP678026	2	669	[26]
Iraq (IQ)	Southeastern Iraq	*Carasobarbus luteus* (F)	MZ047970	1	389	Unpublished
Egypt (EG)	Giza Governorate	*Oreochromis niloticus* (F)	MT140101	1	593	[31]
	Northeastern Egypt	*Nile tilapia* (F)	OQ407866	1	620	Unpublished
	Northeastern Egypt	n. a.	OQ380615	1	591	Unpublished
	Northeastern Egypt	*N. tilapia* (F)	PP177452	1	669	Unpublished
Italy (IT)	Bologna, Santerno River	*S. cephalus* (F)	JF718588	1	610	[1]
	Bologna, Santerno River	*S. cephalus* (F)	JF718590	1	620	[1]
	Bologna, Santerno River	*S. cephalus* (F)	JF718593	1	450	[1]
	Bologna, Santerno River	*S. cephalus* (F)	JF718594	1	617	[1]
	Bologna, Santerno River	*Barbus meridionalis* (F)	JF718592	1	345	[1]
	Bologna, Sillaro River	*Barbus barbus* (F)	JF718591	1	622	[1]
	Bologna, Sillaro River	*B. barbus* (F)	JF718595	1	618	[1]
	Pieve di Soligo, Soligo River	*Lepomis gibbosus* (F)	JF718589	1	597	[1]
	Piemonte, Northern Italy	*Cobitis bilineata* (F)	KU236382	1	621	[13]
	Sesto Fiorentino, Tuscany	*Lissotriton vulgaris* (A)	KM518245–KM518254	10	604–619	[2]
Romania (RO)	Tulcea City, Danube Delta	*Scardinius erythrophthalmus* (F)	MK801718	1	620	[21]
	Rosu Lake, Danube Delta	*Perca fluviatilis* (F)	MK801719	1	583	[21]

Legend: F, fish; B, bird; A, amphibian; n. a., data not available; No., number of sequenced flukes. Note: graphical interpretation of data is presented in Figure 3.

### Design of primers for the PCR amplification of *cox*1 in *C. complanatum* from Slovakia and Italy

**Figure 3:**
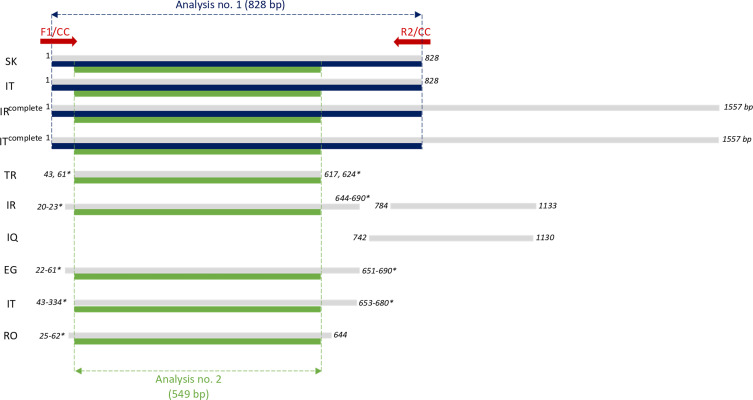
Schematic interpretation of the complete and partial sequences of the mitochondrial *cox*1 gene of *Clinostomum complanatum* from GenBank as summarised in Table 2. Country codes: SK, newly analysed sequences of *C. complanatum* from Slovakia; IT, newly analysed sequences of *C. complanatum* from Italy; ^complete^, complete *cox*1 sequence; IR, Iran; TR, Turkey; IQ, Iraq; EG, Egypt; RO, Romania. Red arrows, annealing positions of primers designed for amplification of partial *cox*1 in samples from Slovakia and Italy; grey lines, schematic regions of *cox*1 genes from GenBank; blue lines, *cox*1 regions selected for analysis no. 1; green lines, *cox*1 regions used for analysis no. 2; numbers, beginnings and ends of the respective sequences; the numbers with asterisks indicate sequences with alternative beginnings and ends.

The graphical interpretation of the *cox*1 data from GenBank pointed out variable sizes and scattered positions of so far analysed *cox*1 regions of different populations (Fig. 3, grey lines). Since the majority of the sequenced *cox*1 fragments were located at the 5′ end of the gene, the 828 bp *cox*1 fragment located at the 5′ end, including the start codon of *cox*1 gene, was selected as the most compatible and comparable region for further studies. The newly designed *C. complanatum*-specific primers for PCR amplification of the selected *cox*1 region were: forward F1/CC (5′-GAGTAAGGGTTATGTTGATTGGG-3′) and reverse R2/CC (5′-CCCAACCATAAACATATGATG-3′) (see red arrows in Fig. 3).

### DNA isolation, PCR amplification, sequencing, and sequence analysis

Genomic DNA was extracted from *C. complanatum* metacercariae using a QIAamp^®^ DNA mini kit (QIAGEN, Hilden, Germany), according to the manufacturer’s protocol. The DNA was stored in deionised water at −20 °C. PCR amplification of the selected 828 bp *cox*1 fragment with the originally designed F1/CC and R2/CC primers was performed in 20 μL reaction mixtures containing 10 ng of DNA template, 1x PCR Master Mix (Fermentas Life Sciences Waltham, MA, USA), and 20 pmol of each primer. After an initial denaturation at 95 °C for 5 min, the samples were subjected to 29 cycles of denaturation at 95 °C for 1 min, annealing at 50 °C for 1 min, and extension at 72 °C for 2 min. After a final extension at 72 °C for 10 min, samples were cooled to 12 °C. The PCR products were visualised on 1% agarose gel and purified using an ExoProStar^TM^ 1-STEP Kit (Illustra, Chicago, IL, USA). Each PCR product was sequenced from both sides using F1/CC forward and R2/CC reverse primers. Sequencing was performed using an Automatic Genetic Analyser 3130xl and a BigDye Terminator v.3.1 Cycle sequencing kit (Applied Biosystems, Foster City, CA, USA). The chromatograms of the sequences were manually trimmed and assembled using Geneious software (version 10.0.5, Biomatters, Auckland, New Zealand). Two independent sets of raw sequence data were checked and aligned to obtain the final contiguous sequences.

### Design of the *cox*1 analyses of *C. complanatum*

The different sizes and scattered positions of the previously analysed *cox*1 regions of *C. complanatum* (Fig. 3) did not allow a single comprehensive analysis including new *cox*1 sequences from Slovakia and Italy and all data retrieved from GenBank. Therefore, two analyses were designed and performed in the present study. Analysis no. 1 (Fig. 3, blue lines) was based on the 828 bp *cox*1 fragment and aimed to assess a genetic variation within and between the currently analysed flukes from Slovakia and Italy. In addition, these populations were analysed and compared with previously published data on *cox*1 of *C. complanatum* from Iran (OP681143) and Italy (MK814187). Analysis no. 2 (Fig. 3, green lines) was based on the 549 bp *cox*1 region at the 5′ end of the gene and included a geographically broader range of *C. complanatum* populations from Europe (Slovakia and Italy, current data; Italy, MK814187, JF718588–JF718595, KU236382, KM518245–KM518254; Romania, MK801718–MK801719), the Middle East (Iran, OP681143, OP709260–OP709261, OP984764, OP678025–OP678026), Turkey (MF928768–MF928774, MT602068) and North Africa (Egypt, MT140101, OQ407866, OQ380615, PP177452).

### Statistics and haplotype network of mitochondrial *cox*1

The programme DnaSP 6 [30] was used to estimate the number of segregating sites, the number of parsimony-informative sites, genetic diversity, haplotype diversity (Hd), nucleotide diversity (Pi), and neutrality test statistics (Fu and Li’s F*, Tajima’s D, Ramos-Onsins and Rozas’ R2 and Raggedness index). The significance of all tests was determined by 10,000 coalescent simulations. The statistical parameters were calculated independently for the newly analysed populations from Slovakia and Italy. Genealogical information of *C. complanatum* populations from Slovakia and Italy along with flukes from other countries (GenBank data) was visualised by haplotype networks using partial *cox*1 sequences in PopArt [19] with the TCS 1.21 algorithm [4].

### Principal components analysis (PCA) and Mantel tests

The statistical analyses were conducted in R, version 4.3.1 [29]. Data manipulations and visualisations were performed using the R package collection – tidyverse [34]. The following packages were used for data analysis: geosphere [16], Biostrings [28], DECIPHER [37], ade4 [7], and vegan [27]. Reproducibility was ensured by setting the random seed to 680. DNA sequences were imported into the R environment using the Biostrings package. Metadata, including latitude and longitude coordinates, were read from the Excel files. Pairwise geographic distances between samples were calculated using the haversine formula via the distHaversine function from the geosphere package, implemented through a custom function which produced distance matrices representing the geographic distances in kilometres between all sample pairs. Genetic similarity matrices were computed using the DistanceMatrix function from the DECIPHER package.

Mantel tests were performed using the mantel function from the vegan package to assess the correlation between genetic similarity and geographical distance. Significance was determined using permutation tests with 999 permutations. The PCA analyses were conducted on the genetic similarity matrices to visualise genetic variation among samples without a priori grouping. The analysis was performed using the dudi.pca function from the ade4 package. PCA scores were extracted and merged with metadata for plotting. The PCA results were visualised using the ggplot2 package from the tidyverse packages collection. Two individual analyses were conducted. Analysis no. 1 was based on the 828 bp *cox*1 fragment and included populations from Slovakia and Italy (original sequences) and data from GenBank (Iran and Italy). Analysis no. 2 was based on the 549 bp *cox*1 region and involved *C. complanatum* populations from Europe (Slovakia, Italy, and Romania), the Middle East (Iran and Turkey) and North Africa (Egypt). See section Design of the *cox*1 analyses of *C. complanatum* for more details.

## Results

### Structure and diversity of mitochondrial haplotypes of *C. complanatum* from Slovakia and Italy

**Supplementary Figure 1:**
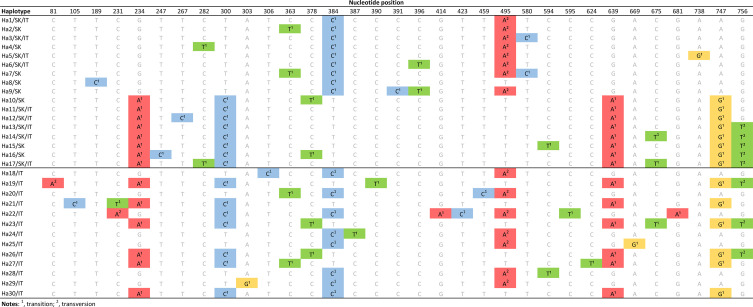
Summary of mutations in mitochondrial *cox*1 haplotypes (CO1-Ha) in *Clinostomum complanatum* from Slovakia (SK) and Italy (IT).

Seventeen mitochondrial *cox*1 haplotypes (CO1-Ha1–CO1-Ha17; 98.7–99.9% similarity) were detected in 55 individuals from Slovakia. The sequences were deposited in GenBank and their accession numbers are listed in Table 3. The dominant haplotype Ha1 was detected in 13 individuals and the second most numerous Ha14 was validated in 11 samples. The majority of single nucleotide point mutations were transitions, while two transversions were present at positions 495 and 756 (Supplementary Fig. 1, see upper index ^2^). All mutations were silent and did not alter the amino acid sequence of the protein.

**Table 3:** Summary of mitochondrial *cox*1 haplotypes (CO1-Ha) of *Clinostomum complanatum* from Slovakia.

Locality	Haplotype	No. of samples	GenBank Acc. No.
**Starohájske river arm (SK-ST)**	CO1-Ha1	3	PQ396177–PQ396179
	CO1-Ha13	1	PQ416077
	CO1-Ha15	1	PQ416099
		**∑ 5**	
**Karloveské river arm (SK-KA)**	CO1-Ha8	2	PQ416065–PQ416066
	CO1-Ha17	2	PQ416108–PQ416109
		**∑ 4**	
**Jarovecké river arm (SK-JA)**	CO1-Ha1	2	PQ406660–PQ406661
	CO1-Ha3	1	PQ406670
	CO1-Ha4	1	PQ400071
	CO1-Ha5	1	PQ403664
	CO1-Ha6	1	PQ416014
	CO1-Ha10	1	PQ416068
	CO1-Ha11	1	PQ416072
	CO1-Ha13	2	PQ416078–PQ416079
	CO1-Ha14	1	PQ416086
		**∑ 11**	
**Biskupické river arm (SK-BI)**	CO1-Ha1	7	PQ406662–PQ406668
	CO1-Ha2	1	PQ399670
	CO1-Ha3	1	PQ406671
	CO1-Ha7	1	PQ416064
	CO1-Ha10	1	PQ416069
	CO1-Ha11	1	PQ416073
	CO1-Ha12	2	PQ416074–PQ416075
	CO1-Ha13	1	PQ416080
	CO1-Ha14	8	PQ416087–PQ416094
	CO1-Ha16	1	PQ416106
		**∑ 24**	
**Šulianske Lake (SK-SL)**	CO1-Ha1	1	PQ406669
	CO1-Ha6	2	PQ416015–PQ416016
	CO1-Ha9	1	PQ416067
	CO1-Ha10	2	PQ416070–PQ416071
	CO1-Ha12	1	PQ416076
	CO1-Ha13	2	PQ416081–PQ416082
	CO1-Ha14	2	PQ416095–PQ416096
		**∑ 11**	
**Total**	**CO1-Ha1–17**	**55**	

**Figure 4:**
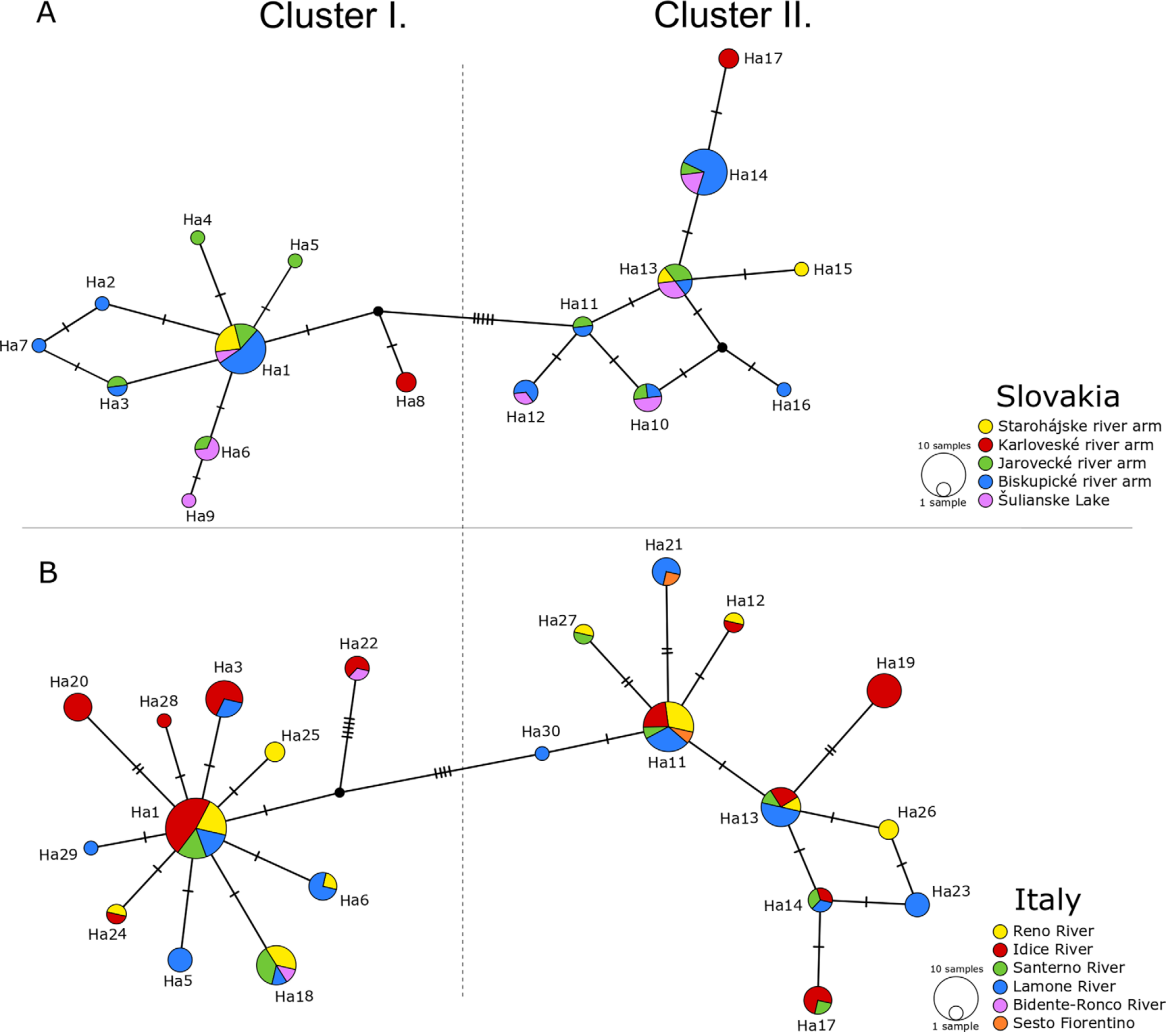
Haplotype network diagram of analysis no. 1 (828 bp; Fig. 3, blue lines) based on mitochondrial *cox*1 haplotypes of *Clinostomum complanatum* from (A) Slovakia; (B) Italy. The size of the haplotypes is proportional to the number of samples. Each hatch mark represents a single mutation, while black dots symbolize missing or unsampled haplotype. Details on haplotype numbers are given in Table 3 (Slovakia) and Table 4 (Italy).

The haplotype network of *C. complanatum* from Slovakia revealed the presence of two clusters. Haplotypes Ha1–9 shared several common mutations (Supplementary Fig. 1) and formed cluster I, while Ha10–17 formed cluster II, which was separated from cluster I by five mutations (Fig. 4A). Cluster I was characterised by a star-like pattern with Ha1 as the central haplotype and Ha2–9 placed on the individual mutation pathways separated from the central haplotype by 1–3 mutations. Cluster II was represented by a diffuse haplotype network. Genetic structuring associated with the particular locality was not detected (Fig. 4A).

Twenty-two haplotypes were identified in 102 samples from Italy. The sequences were deposited in GenBank and their accession numbers are presented in Table 4. Nine haplotypes were common to samples from Italy and Slovakia (Ha1, 3, 5, 6, 11, 12, 13, 14, and 17) and 13 haplotypes were specific to Italy (Ha18–30). The dominant haplotype Ha1 (identical to the Slovak population) was detected in 19 individuals, and the second most numerous haplotype Ha11 was found in 13 individuals. The majority of single nucleotide point mutations were transitions, while four transversions occurred at positions 81, 231, 495, and 756 (Supplementary Fig. 1, see upper index ^2^). All mutations were silent and did not alter the amino acid sequence of the protein.

**Table 4:** Summary of mitochondrial *cox*1 haplotypes (CO1-Ha) of *Clinostomum complanatum* from Italy.

Locality	Haplotype	No. of samples	GenBank Acc. No.
**Idice River (IT-ID)**	CO1-Ha1	9	PQ461981–PQ461989
	CO1-Ha3	5	PQ462000–PQ462004
	CO1-Ha11	3	PQ462014–PQ462016
	CO1-Ha12	1	PQ462027
	CO1-Ha13	2	PQ462029–PQ462030
	CO1-Ha14	1	PQ462037
	CO1-Ha17	3	PQ462040–PQ462042
	CO1-Ha19	6	PQ458471–PQ458476
	CO1-Ha20	4	PQ458533–PQ458536
	CO1-Ha22	2	PQ458557–PQ458558
	CO1-Ha24	1	PQ458564
	CO1-Ha28	1	PQ458631
		**∑ 38**	
**Santerno River (IT-SA)**	CO1-Ha1	3	PQ461990–PQ461992
	CO1-Ha11	1	PQ462017
	CO1-Ha13	1	PQ462031
	CO1-Ha14	1	PQ462038
	CO1-Ha17	1	PQ462043
	CO1-Ha18	3	PQ458477–PQ458479
	CO1-Ha27	1	PQ458598
		**∑ 11**	
**Bidente-Ronco (IT-BI)**	CO1-Ha22	1	PQ458559
	CO1-Ha18	1	PQ458480
		**∑ 2**	
**Reno River (IT-RE)**	CO1-Ha1	4	PQ461993–PQ461996
	CO1-Ha6	1	PQ462010
	CO1-Ha11	4	PQ462018–PQ462021
	CO1-Ha12	1	PQ462028
	CO1-Ha13	1	PQ462032
	CO1-Ha18	3	PQ458481–PQ458483
	CO1-Ha24	1	PQ458565
	CO1-Ha25	2	PQ458595–PQ458596
	CO1-Ha26	2	PQ458593–PQ458594
	CO1-Ha27	1	PQ458599
		**∑ 20**	
**Lamone River (IT-LA)**	CO1-Ha1	3	PQ461997–PQ461999
	CO1-Ha3	2	PQ462005–PQ462006
	CO1-Ha5	3	PQ462007–PQ462009
	CO1-Ha6	3	PQ462011–PQ462013
	CO1-Ha11	4	PQ462022–PQ462025
	CO1-Ha13	4	PQ462033–PQ462036
	CO1-Ha14	1	PQ462039
	CO1-Ha18	1	PQ458484
	CO1-Ha21	3	PQ458540–PQ458542
	CO1-Ha23	3	PQ458560–PQ458562
	CO1-Ha29	1	PQ458630
	CO1-Ha30	1	PQ458632
		**∑ 29**	
**Sesto Fiorentino (IT-SF)**	CO1-Ha11	1	PQ462026
	CO1-Ha21	1	PQ458543
		**∑ 2**	
**Total**	**CO1-Ha1, 3, 5, 6, 11–14, 17**	**102**	
	**CO1-Ha18–30**		

The haplotype network of *C. complanatum* from Italy showed the presence of two clusters (Fig. 4B), with topology resembling that of *C. complanatum* from Slovakia (Fig. 4A). Cluster I. had a satellite-like structure with Ha1 as the central haplotype. Nine haplotypes were placed on the mutation pathways separated from the central haplotype by 1–2 mutations. Cluster II displayed a diffuse structure, similar to the population from Slovakia. Genetic structuring associated with the particular locality was not detected (Fig. 4B).

### Interrelationships of different populations of *C. complanatum*

**Figure 5:**
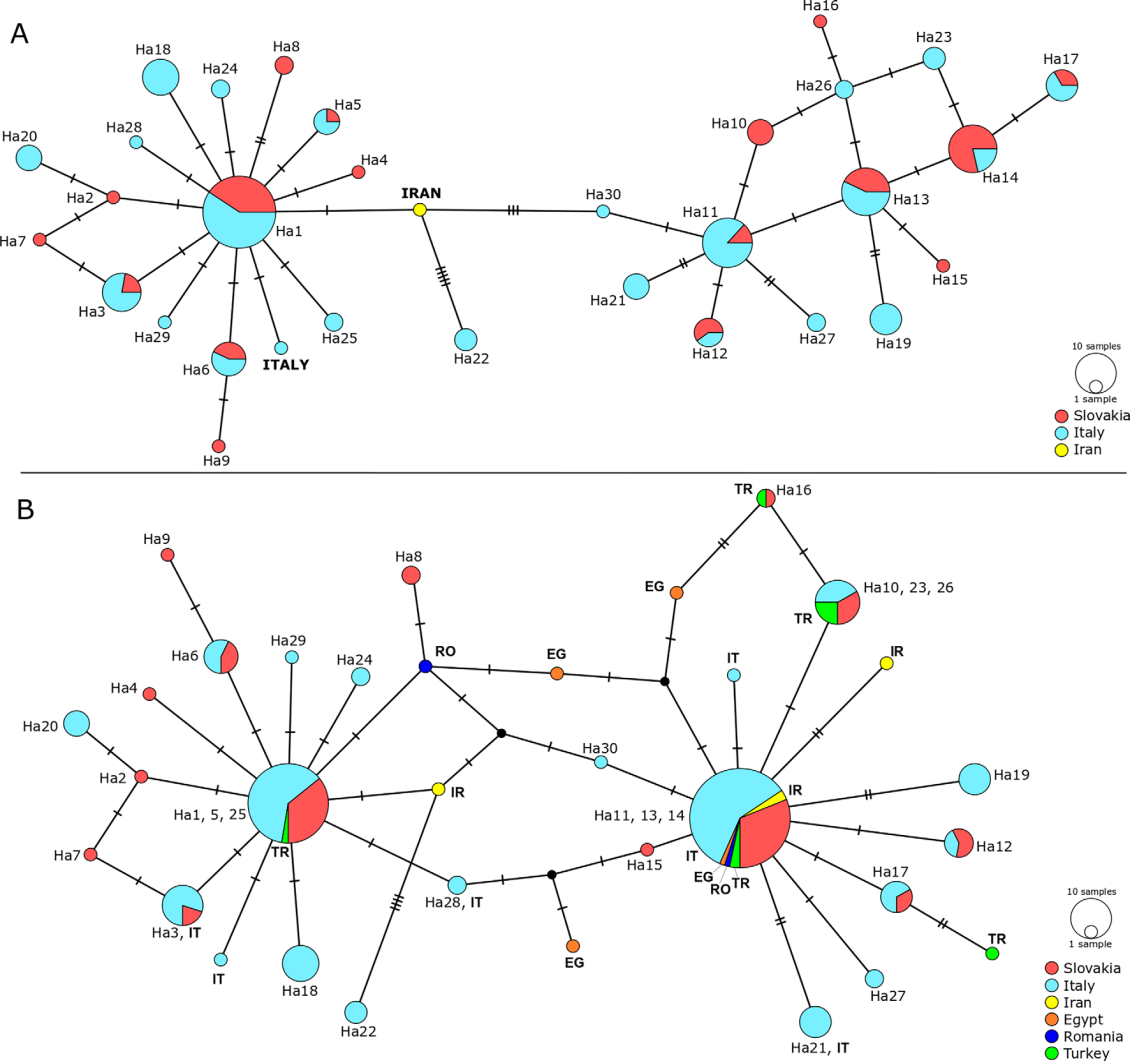
Haplotype network diagram of (A) analysis no. 1 (828 bp; Fig. 3, blue lines) based on *cox*1 haplotypes of *Clinostomum complanatum* from Slovakia/SK, Italy/IT, and Iran/IR; (B) analysis no. 2 (549 bp; Fig. 3, green lines) based on mitochondrial *cox*1 haplotypes of *C. complanatum* from SK, IT, IR, Turkey/TR, Egypt/EG and Romania/RO. The size of the haplotypes is proportional to the number of samples. Each hatch mark represents a single mutation, while black dots symbolise missing or unsampled haplotypes. Originally analysed haplotypes are displayed by haplotype numbers, data from GenBank are displayed by country names (Fig. 5A) or country codes (Fig. 5B). Details on haplotype numbers are given in Tables 3 and 4.

The haplotype network based on the 828 bp *cox*1 region (Fig. 3, blue lines) of the originally analysed 157 individuals from Slovakia and Italy and GenBank sequences of flukes from Iran and Italy is presented in Figure 5A. It revealed a genetic architecture of flukes similar to that from Slovakia and Italy and obvious genetic exchange between both populations, as it shows a similar topology of two evident clusters. The central haplotype Ha1 of cluster I was shared by samples from both countries, similar to the most numerous haplotypes Ha11, 13, 14, and 17 of cluster II. The *cox*1 data of samples from Italy and Iran retrieved from GenBank were placed on separate mutation pathways within cluster I and were each separated from the central Ha1 by a single mutation.

The haplotype network (Fig. 5B) based on the analysis no. 2 (Fig. 3, green lines) shows that samples from Europe (Italy, Romania, and Turkey), North Africa (Egypt) and the Middle East (Iran) possessed haplotypes identical to the central haplotypes Ha1/5/25 and Ha11/13/14, implying genetic admixture of flukes from remote localities. In addition, samples from Iran, Romania, Egypt, and Turkey were placed on separate mutation pathways localised within both clusters. No distinct genetic structure was observed in either individuals from southern Europe or samples from the Middle East.

### Statistical analyses of mitochondrial data

The genetic diversity of the newly analysed populations of *C. complanatum* from Slovakia and Italy was quite similar (Table 5). The population from Italy showed slightly higher genetic diversity across all metrics than the population from Slovakia, which may be due to the unequal number of samples (55 for Slovakia *vs.* 102 for Italy). The data set from Slovakia contained 19 segregating sites, of which 15 were parsimony-informative, while the population from Italy had 30 segregating sites, of which 28 were parsimony-informative. The Hd and Pi diversity levels were comparable between the two populations, with Italy showing slightly higher values (Hd = 0.926 for Italy *vs.* 0.890 for Slovakia; Pi = 0.00629 for Italy *vs.* 0.00577 for Slovakia).

**Table 5:** Molecular variability and statistical tests of neutrality of the mitochondrial *cox*1 haplotypes of *Clinostomum complanatum* from Slovakia and Italy.

Parameter/tests	Slovakia	Italy
N	55	102
S	19	30
PIS	15	28
H	17	22
Hd	0.890	0.926
Pi	0.00577	0.00629
Fu and Li’s F*	0.2507 (*p* = 0.61300)	0.7521 (*p* = 0.79600)
Tajima’s D	0.4706 (*p* = 0.73900)	−0.3878 (*p* = 0.40460)
Ramos-Onsins and Rozas’ R2	0.1227 (*p* = 0.72850)	0.0862 (*p* = 0.45980)
Raggedness statistics	0.0420 (*p* = 0.40640)	0.0228 (*p* = 0.18180)

N, number of analysed samples; S, number of segregating sites; PIS, number of parsimony-informative sites; H, number of haplotypes; Hd, haplotype diversity; Pi, nucleotide diversity.

In *C. complanatum* from Slovakia, a possible balancing selection in Fu and Li’s F* and Tajima’s D was observed, but the *p*-values showed no significant deviation from neutrality. Ramos-Onsins and Rozas’ R2 and Raggedness statistics also showed no evidence of significant population changes in the Slovak population, and with *p*-values over 0.05, the results were not statistically significant.

In the population of *C. complanatum* from Italy, the positive value of Fu and Li’s F* (0.7521) indicated balancing selection, while the negative value of Tajima’s D (−0.3878) suggested an excess of low-frequency genetic variants that could be associated with purifying selection or population expansion. Nevertheless, no significant deviation from neutrality was observed, and the absence of significant population changes was also confirmed by Ramos-Onsins and Rozas’ R2 and Raggedness statistics.

Despite the different sample sizes, the overall genetic diversity in the populations from Slovakia and Italy was similar, and the neutrality tests revealed no clear evidence of significant population changes. Samples of *C. complanatum* from other countries (GenBank data) could not be statistically evaluated due to the low number of individuals within the respective population in each country.

**Figure 6:**
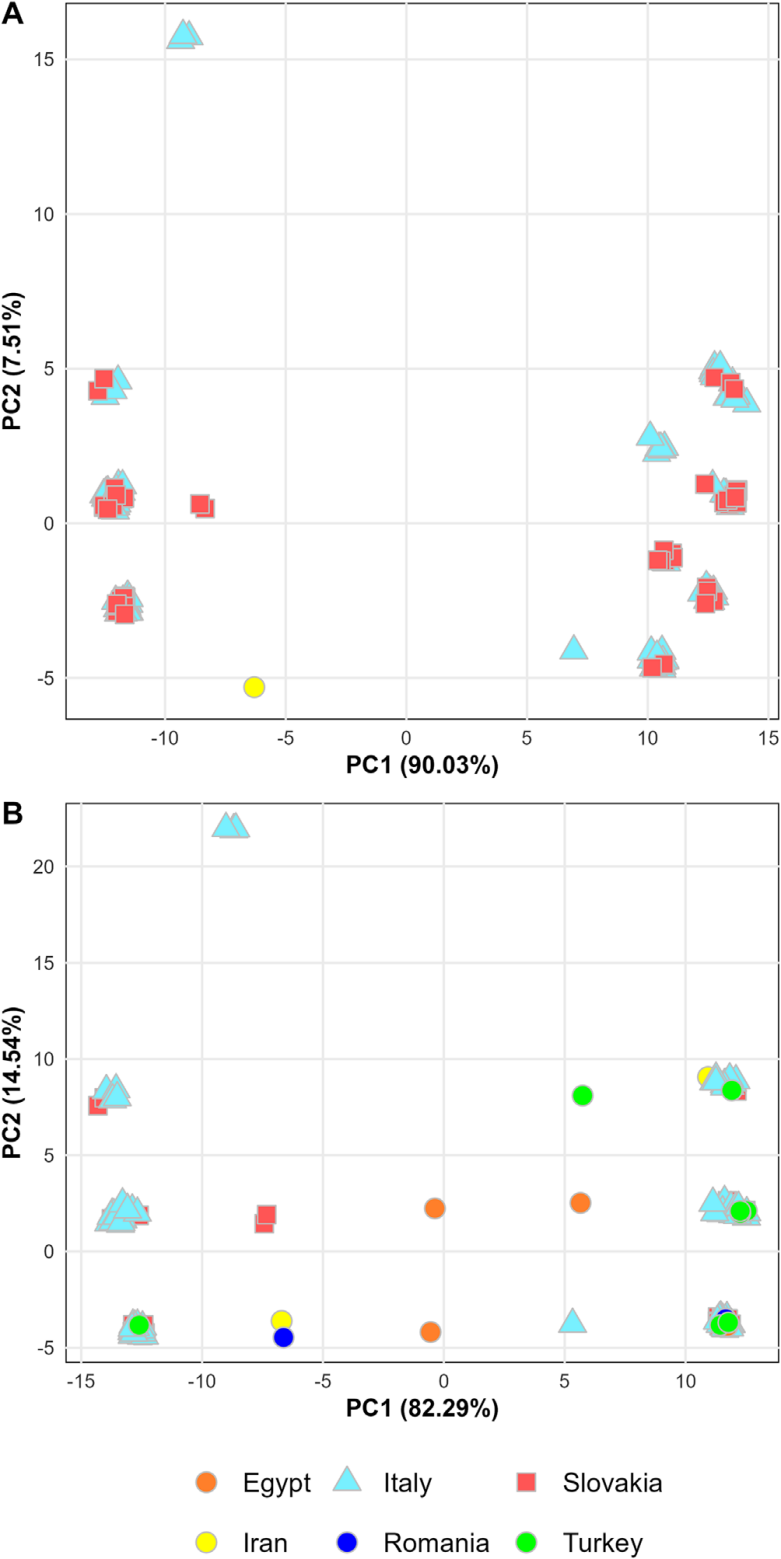
Principal component analysis (PCA)-based visualisation of cox1 genetic variation among *Clinostomum complanatum* from (A) Slovakia, Italy, and Iran (analysis no. 1; 828 bp; Fig. 3, blue lines) and (B) Slovakia, Italy, Iran, Romania, Egypt, and Turkey (analysis no. 2; 549 bp; Fig. 3, green lines).

The Mantel test of the analysis no. 1 revealed a weak but statistically significant positive correlation between genetic similarity and geographic distance (Mantel statistic *r* = 0.0374; *p* = 0.008). In contrast, the analysis based on the 549 bp dataset showed no significant correlation (Mantel statistic *r* = 0.0144). Principal components analyses for both datasets (Figs. 6A, 6B) did not reveal any clustering based on geographic origin. Samples from different countries were intermixed in the PCA plots, as colouring by country did not reveal any patterns associated with the region of origin.

## Discussion

The genetic structure of *C. complanatum* from five sampling sites on the Danube River in south-west Slovakia was represented by a great number of *cox*1 haplotypes. A similar genetic architecture and high heterogeneity were observed in the population of *C. complanatum* from five rivers of the Emilia-Romagna region in Italy. The results indicate that *C. complanatum* has established stable populations in both localities.

The Danube floodplain forests, which extend around the Danube River and its tributaries, are a complex and dynamic hydrological system. They provide suitable biological conditions for all intermediate (snails, fishes, and amphibians) and definitive hosts (piscivorous birds) involved in the life cycle of *C. complanatum*. The seasonal changes in the water level of the Danube River are a strong ecological factor influencing the bird populations [10]. Rising water levels in spring or during flood events may have a positive effect on the diversity and abundance of birds [10], whose infection is positively correlated with the intensity of parasitic infection in intermediate hosts [6, 15].

The Protected Bird Area within Danube floodplain forests is internationally recognised as an important breeding and nesting area, refuge, migration corridor, and wintering area for numerous native and migratory aquatic birds (*e.g.* herons, cormorants, divers, grebes, pelicans, storks, rails, ibises, spoonbills, flamingos, ducks, swans, geese, cranes, shorebirds, gulls, and terns) [25]. These birds depend on wetlands for at least part of their annual cycle (https://www.worldmigratorybirdday.org/african-eurasian-flyway) and fly from the temperate regions of Europe to Africa and Asia (https://datazone.birdlife.org/sowb/spotflyway).

Flukes from Italy originated from fish from the Emilia-Romagna region, which hosts breeding populations of several bird species. Of these, the grey heron *Ardea cinerea*, the little egret *Egretta garzetta*, and the night heron *Nycticorax nycticorax*, the common bird hosts of *C. complanatum*, are the most abundant aquatic birds in the region [12]. In addition, the Italian coastal wetlands in the upper part of the Adriatic Sea and inlands along the Po River, the longest river in Italy, provide particularly rich foraging grounds for breeding populations of herons and egrets [11].

The presence of common haplotypes and a similar topology of the haplotype networks of *C. complanatum* from Slovakia and Italy indicate genetic exchange between these populations. In addition, the haplotypes of samples from distant localities, namely Turkey, Egypt and Iran, showed possible gene flow among the populations across wide geographical regions – from Central Europe down to the Mediterranean region, North Africa, and the Middle East. The genetic homogeneity of *C. complanatum* can be linked to the distribution, biology, and migratory routes of their bird hosts.

Aquatic birds may connect wetlands separated by hundreds of kilometres, contributing to the maintenance of biodiversity [33], promoting gene flow among populations, and influencing the population and community composition, structure and dynamics [5]. Migratory piscivorous birds, mainly the grey heron, the purple heron, the night heron, and the great cormorant are apparently the most important long-distance transmitters of *C. complanatum*. Their elevated mobility enables them to spread eggs and transmit the parasite over large areas and between continents.

The populations of the great heron are either sedentary, or dispersive and migratory, depending on the region. Some eastern European populations migrate towards south or south-east and reach sub-Saharan Africa in winter [23]. Populations of the purple heron from the western Palaearctic are migratory, wintering in the Mediterranean Basin, the Middle East, and Africa [24]. As with the night heron, the western Palaearctic populations migrate across the Mediterranean and the Sahara, reaching tropical Africa. Some populations overwinter in the Mediterranean Basin, while eastern European birds may migrate to the Middle East and the Indian subcontinent [17]. Several subspecies of the great cormorant have been recognised. The subspecies *Phalacrocorax carbo sinensis* from northern Eurasia is a migratory bird; many birds from northern Europe winter in the Mediterranean region, some also in North Africa and the Middle East and reach the Persian Gulf [14].

The regular seasonal migrations of herons and cormorants, and possibly other bird hosts of *C. complanatum*, have resulted in transmission of the fluke throughout the Palaearctic region. Common mitochondrial haplotypes detected in flukes from Europe, Egypt, and Iran indicate that migratory birds help to maintain the genetic homogeneity of *C. complanatum* by crossing geographical barriers between Central Europe, the Mediterranean region, North Africa, and the Middle East.

The absence of genetic structuring associated with geography, as determined in *C. complanatum* from Slovakia and Italy, has previously been observed in other flukes with life cycles involving fish and migratory piscivorous birds as intermediate and definitive hosts, respectively. No geographic structuring was detected in *Diplostomum pseudospathaceum* (Digenea: Diplostomatidae) from eight European countries [9]. Furthermore, a low level of genetic differentiation was detected between populations of *Cryptocotyle lingua* (Digenea: Opisthorchiidae) from geographically distinct areas in the English Channel and the North Sea [8]. In accordance with our conclusions, a high level of dispersal by mobile definitive hosts drives the genetic diversity of populations of mentioned flukes across Europe, minimising the effect of significant genetic drift [9]. However, the presence of two clearly different clusters of *cox*1 haplotypes in populations from Slovakia and Italy was not observed in the abovementioned trematode species, whose haplotype networks displayed either typical star-shaped structure or more complex network topology [8, 9].

**Supplementary Figure 2:**
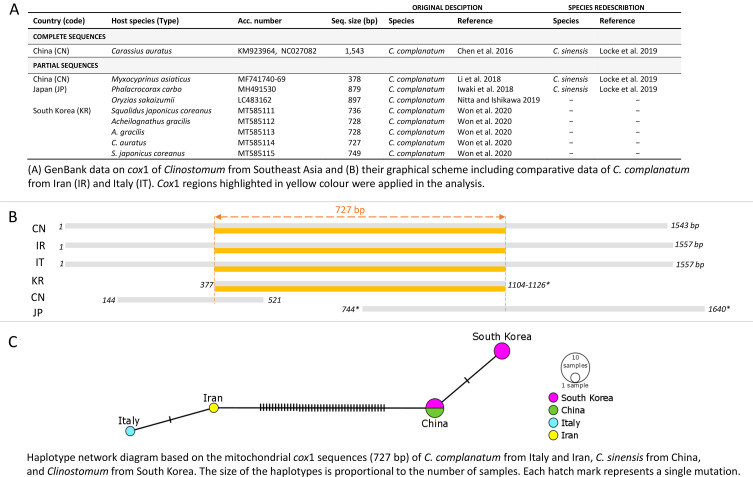
Mitochondrial *cox*1 data and analysis of *Clinostomum sinensis* and *Clinostomum complanatum*.

Species of the genus *Clinostomum* from Southeast Asia evidently deserve further detailed studies and require taxonomic revisions and redescriptions. Taxonomy of parasites initially identified as *C. complanatum* in several Southeast Asian countries has been mostly based on their morphology (Supplementary Table 2), while the mitochondrial *cox*1 data are available only for flukes from South Korea [36] (Supplementary Figs. 2A, 2B). The analysis based on the 727 bp *cox*1 fragment of comparative samples of *C. complanatum* (Italy, MK814187 and Iran, OP681143), sequences of *C. sinensis* from China (KM923964, NC027082), and five sequences of flukes initially identified as *C. complanatum* from South Korea (MT585111–MT585115) (Supplementary Fig. 2B, yellow lines) resulted in a linear pattern of the haplotype network, with two distinct clusters (Supplementary Fig. 2C). The first one included *C. complanatum* from Europe and the Middle East (Italy and Iran), while the second cluster included *C. sinensis* from China and samples from South Korea, indicating that flukes from South Korea are *C. sinensis.*

## Conclusion

The results of the mitochondrial *cox*1 analyses revealed that *C. complanatum* from Slovakia and Italy are stable populations with high genetic diversity. The haplotypes of samples from other localities showed possible gene flow among the populations from Central Europe down to the Mediterranean region, North Africa, and the Middle East. The currently applied mitochondrial *cox*1 gene has been widely used in molecular taxonomy and population genetics of many taxa, including *Clinostomum*. The numerous *cox*1 data available in GenBank can be used as reference or comparative samples in different taxonomic, phylogenetic, or population studies. However, in this particular case, it was not possible to perform a single comprehensive analysis due to the variable size and scattered positions of the *cox*1 sequences of *C. complanatum* from different localities. To improve the usability of this barcoding tool, future studies on *C. complanatum* should focus on the 5′ end of the *cox*1 gene, for which most sequence data are available.

## Supplementary material

The supplementary material for this article can be found at https://www.parasite-journal.org/10.1051/parasite/2024080/olm. *Supplementary Table 1*: Summary of hosts and localities of *Clinostomum complanatum* in Europe, Turkey, Egypt, and the Middle East. *Supplementary Table 2*: Summary of hosts and localities of *Clinostomum sinensis* (in bold) *and Clinostomum* sp*.* originally described as *Clinostomum complanatum* from Asia.
