# Supplementary material for: Population structure of Clinostomum complanatum (Trematoda: Digenea) with new data on haplotype diversity of flukes from Slovakia and Italy
Source: Parasite. 2025 Jan 22;32:3. doi: 10.1051/parasite/2024080 (PMC11752738; doi:10.1051/parasite/2024080)
Supplement: Supplementary file 1 — Supplementary Table 1: Summary of hosts and localities of Clinostomum complanatum in Europe, Turkey, Egypt, and the Middle East. [file parasite-32-3-s1.pdf]

Supplementary Table 1. Summary of hosts and localities of *Clinostomum complanatum* in Europe, Turkey, Egypt, and the Middle East.

CONTINENT/REGION<br>Country (country code)<br>Locality	Type of host	Family	Host species	Common name	P (%)	Reference
**EUROPE**						
**Italy (IT)**						
Bologna Province, Emilia-Romagna Region	F	Cyprinidae	*Barbus barbus*	barbel	n. a.	Andreucci et al. 1994
Sillaro River, Emilia-Romagna Region	F	Cyprinidae	*B. barbus*	barbel	n. a.	Caffara et al. 2011
Santerno River, Emilia-Romagna Region	F	Cyprinidae	*Barbus meridionalis*	Mediterranean barbel	n. a.	Caffara et al. 2011
Bologna Province, Emilia-Romagna Region	F	Cyprinidae	*Squalius cephalus*	European chub	n. a.	Andreucci et al. 1994
Soligo River, Veneto Region	F	Cyprinidae	*S. cephalus*	European chub	n. a.	Caffara et al. 2011
Santerno River, Emilia-Romagna Region	F	Cyprinidae	*S. cephalus*	European chub	n. a.	Locke et al. 2019
Pordenone, Friuli Venezia Giulia Region	F	Cyprinidae	*Telestes muticellus*	Italian vairon	25.0	Dorigo et al. 2023
Piemonte Region	F	Cobitidae	*Cobitis bilineata*	Italian spined loach	40.0	Gaglio et al. 2016
Pordenone, Friuli Venezia Giulia Region	F	Cobitidae	*C. bilineata*	Italian spined loach	72.0	Dorigo et al. 2023
Vercelli Province, Piedmont Region	F	Cobitidae	*Cobitis taenia*	spined loach	n. a.	Maccagno 1934
Endine Lake, Lombardy, Bergamo Province	F	Percidae	*Perca fluviatilis*	European perch	18.7	Menconi et al. 2020
Vercelli Province, Piedmont Region	B	Ardeidae	*Ardea cinerea*	grey heron	n. a.	Maccagno 1934
Bologna Province, Emilia-Romagna Region	B	Ardeidae	*A. cinerea*	grey heron	n. a.	Andreucci et al. 1994
n. a.	B	Ardeidae	*A. cinerea*	grey heron	n. a.	Caffara et al. 2011
n. a.	B	Ardeidae	*Ardea purpurea*	purple heron	n. a.	Caffara et al. 2011
Bologna Province, Emilia-Romagna Region	B	Ardeidae	*A. purpurea*	purple heron	n. a.	Andreucci et al. 1994
n. a.	B	Ardeidae	*Egretta garzetta*	little egret	n. a.	Caffara et al. 2011
Sesto Fiorentino, Tuscany	A	Salamandridae	*Lissotriton vulgaris*	smooth newt	n. a.	Caffara et al. 2014
Sesto Fiorentino, Tuscany	A	Salamandridae	*Triturus carnifex*	Italian crested newt	n. a.	Caffara et al. 2014
**Slovakia (SK)**						
Tisa River Basin	F	Cyprinidae	*Carassius auratus*	goldfish	57.9	Oros and Hanzelová 2009
Danube River Basin	F	Cyprinidae	*Rhodeus amarus*	European bitterling	3.7	Dávidová et al. 2008
Okna River	F	Cobitidae	*Cobitis elongatoides*	–	19.4-70.0	Fedorčák et al. 2019
Turňa River	F	Cobitidae	*C. elongatoides*	–	81.3	Fedorčák et al. 2019
Danube River Basin	F	Cobitidae	*Cobitis* sp.	–	n. a.	Žitňan 1979
Tisa River Basin	F	Cobitidae	*C. taenia*	spined loach	29.6	Oros and Hanzelová 2009
Tisa River Basin	F	Cobitidae	*Misgurnus fossilis*	weatherfish	1.0	Oros and Hanzelová 2009
Danube River Basin	F	Percidae	*P. fluviatilis*	European perch	7.7	Juhásová et al. 2019
Danube River Basin	B	Ardeidae	*A. purpurea*	purple heron	n. a.	Macko 1960
**Romania (RO)**						
Motru River	F	Cyprinidae	*Gobio* sp.	–	n. a.	Cojocaru 2009
Motru River	F	Cyprinidae	*Rutilus* sp.	–	n. a.	Cojocaru 2009
Channel 36 near Tulcea, Danube Delta	F	Cyprinidae	*Scardinius erythropthalmus*	common rudd	n. a.	Locke et al. 2019
Motru River	F	Cyprinidae	*Squalius* sp.	–	n. a.	Cojocaru 2009
Preajba River	F	Percidae	*P. fluviatilis*	European perch	n. a.	Goga and Codreanu-Balcescu 2011
Rosu Lake, Danube Delta	F	Percidae	*P. fluviatilis*	European perch	n. a.	Locke et al. 2019

**Poland (PL)**						
Lichenskie Lake, Konin County	F	Cyprinidae	*Rutilus rutilus*	roach	n. a.	Grabda-Kazubska 1974
Lichenskie Lake, Konin County	F	Percidae	*P. fluviatilis*	European perch	n. a.	Grabda-Kazubska 1974
Masurian Lake Land	B	Ardeidae	*A. cinerea*	grey heron	n. a.	Grabda-Kazubska 1974
**Croatia (CR)**						
Orljava River, Sava River Basin	F	Cyprinidae	*Cyprinus carpio*	common carp	5.9	Gjurčević et al. 2022
Orljava River, Sava River Basin	F	Cyprinidae	*S. cephalus*	European chub	23.4	Gjurčević et al. 2022
**Czech Republic (CZ)**						
Morava River Basin	F	Cyprinidae	*Rhodeus amarus*	European bitterling	8.0-23.0	Kadlec et al. 2003
Morava River Basin	F	Percidae	*P. fluviatilis*	European perch	8.0-23.0	Kadlec et al. 2003
**France (FR)**						
Durance River, Rhône River Basin	F	Cyprinidae	*R. amarus*	European bitterling	8.3	Dávidová et al. 2008
**Hungary (HU)**						
Tisa and Zala Rivers	F	Odontobutidae	*Perccottus glenii*	Amur sleeper	0.6	Antal et al. 2015
**Moldova (MO)**						
Draghişte River, Raionul Edineţ	F	Percidae	*P. fluviatilis*	European perch	n. a.	Gologan 2020
**Serbia (RS)**						
Danube River Basin	F	Percidae	*P. fluviatilis*	European perch	n. a.	Djikanović et al. 2011
**Ukraine (UA)**						
Tisa River Basin	F	Cobitidae	*C. elongatoides*	–	77.4	Fedorčák et al. 2019
**EUROPE/ASIA**						
**Turkey (TR)**						
Southern Turkey	F	Cyprinidae	*Garra rufa*	red garra	90.0	Cagatay et al. 2022
Gala Lake, Edirne Province	F	Cyprinidae	*R. rutilus*	roach	7.7	Soylu 2013
Gala Lake, Edirne Province	F	Cyprinidae	*S. erythrophthalmus*	common rudd	17.2	Soylu 2013
Central Anatolia Region	F	Cyprinidae	*S. cephalus*	European chub	2.4	Simsek et al. 2018
Sığırcı Lake, Edirne Province	F	Centrarchidae	*Lepomis gibbosus*	pumpkinseed	8.3	Çolak 2013
Sığırcı Lake, Edirne Province	F	Percidae	*P. fluviatilis*	European perch	13.1	Çolak 2013
Sığırcı Lake, Edirne Province	F	Percidae	*P. fluviatilis*	European perch	15.4	Soylu 2013
Gala Lake, Edirne Province	F	Percidae	*P. fluviatilis*	European perch	53.8	Soylu 2013
Sığırcı Lake, Edirne Province	F	Percidae	*Sander lucioperca*	zander	8.3	Çolak 2013
**NORTH AFRICA**						
**Egypt (EG)**						
Nile River	F	Clariidae	*Clarias lazera*	African catfish	n. a.	El-Shahawy et al. 2017
Nile River	F	Cichlidae	*Oreochromis niloticus*	Nile tilapia	n. a.	El-Shahawy et al. 2017
North Giza	F	Cichlidae	*O. niloticus*	Nile tilapia	n. a.	Mahdy et al. 2021
Nile River	F	Latidae	*Lates niloticus*	Nile perch	n. a.	El-Shahawy et al. 2017
Nile River, Qena Province	B	Phalacrocoracidae	*Phalacrocorax carbo*	great cormorant	n. a.	El-Dakhly et al. 2018

**THE MIDDLE EAST**						
**Iran (IR)**						
Shiroud River, Southern Caspian Sea Basin	F	Cyprinidae	*Alburnoides bipunctatus*	schneider	7.9	Aghlmandi et al. 2018
Gheshlagh River, Kurdistan Province	F	Cyprinidae	*Alburnus mossulensis*	Mossul bleak	5.0	Maleki et al. 2018
Gheshlagh River, Kurdistan Province	F	Cyprinidae	*Capoeta damascina*	Levantine scraper	4.1	Maleki et al. 2018
Shiroud River, Southern Caspian Sea Basin	F	Cyprinidae	*Capoeta gracilis*	kapwaeti	24.3	Aghlmandi et al. 2018
Gheshlagh River, Kurdistan Province	F	Cyprinidae	*G. rufa*	red garra	15.0	Maleki et al. 2018
Shiroud River, Southern Caspian Sea Basin	F	Cyprinidae	*S. cephalus*	European chub	16.6	Aghlmandi et al. 2018
Gheshlagh River, Kurdistan Province	F	Cyprinidae	*S. cephalus*	European chub	3.0	Maleki et al. 2018
Mehran River, Hormuzgan Province	F	Aphaniidae	*Aphanius dispar*	Arabian pupfish	4.1	Gholami et al. 2011
Shiroud River, Southern Caspian Sea Basin	F	Cobitidae	*Cobitis cf taenia*	spined loach	4.9	Aghlmandi et al. 2018
Tonekabon, Mazandaran Province	B	Ardeidae	*Nycticorax nycticorax*	night heron	n. a.	Monnens et al. 2023
**Iraq (IQ)**						
Greater Zab River, Kurdistan Region	F	Mastacembelidae	*Mastacembelus mastacembelus*	spiny eel	0.8	Bashě and Abdullah 2010
East Al-Hammar Marsh	B	Ardeidae	*A. cinerea*	grey heron	n. a.	Al-Salim and Ali 2010
East Al-Hammar Marsh	B	Ardeidae	*Ardeola ralloides*	small white bittern	n. a.	Al-Salim and Ali 2010
**Israel (IL)**						
Sea of Galilee (Tiberias Lake)	F	Cyprinidae	*Barbus canis*	barbel	n. a.	Dzikowski et al. 2004
Sea of Galilee (Tiberias Lake)	B	Ardeidae	*E. garzetta*	little egret	n. a.	Dzikowski et al. 2004
Sea of Galilee (Tiberias Lake)	H	–	n. a.	–	–	Witenberg 1944

Legend: F, fish; B, bird; A, amphibian; H, human; P, prevalence; n. a., data not available

REFERENCES

Andreucci A, Canestri Trotti G, Fioravanti ML, Giani G, Nobile L, Restani R, Rizzoli M. 1994. Indagine sulla diffusione di *Clinostomum complanatum* (Trematoda: Digenea) nella ittiofauna della provincia di Bologna. Biologia Oggi, 1-2, 27-32.

Aghlmandi F, Habibi F, Afraii MA, Abdoli A, Shamsi S. 2018. Infection with metacercaria of *Clinostomum complanatum* (Trematoda: Clinostomidae) in freshwater fishes from Southern Caspian Sea Basin. Revue de Médecine Vétérinaire, 169, 147-151.

Al-Salim NK, Ali AH. 2010. First record of three species of trematodes of the genus *Clinostomum* Leidy, 1856 (Digenea: Clinostomidae) parasitic in piscivorous birds from East Al-Hammar Marsh, South of Iraq. Marsh Bulletin, 5, 27-42.

Antal L, Székely C, Molnár K. 2015. Parasitic infections of two invasive fish species, the Caucasian dwarf goby and the Amur sleeper, in Hungary. Acta Veterinaria Hungarica, 63, 472-484.

Bashě SKR, Abdullah SMA. 2010. Parasitic fauna of spiny eel *Mastacembelus mastacembelus* from Greater Zab river in Iraq. Iranian Journal of Veterinary Research, Shiraz University, 11, 30, 18-27.

Caffara M, Locke SA, Gustinelli A, Marcogliese DJ, Fioravanti ML. 2011. Morphological and molecular differentiation of *Clinostomum complanatum* and *Clinostomum marginatum* (Digenea: Clinostomidae) metacercariae and adults. Journal of Parasitology, 97, 884-891.

Caffara M, Bruni G, Paoletti C, Gustinelli A, Fioravanti ML. 2014. Metacercariae of *Clinostomum complanatum* (Trematoda: Digenea) in European newts *Triturus carnifex* and *Lissotriton vulgaris* (Caudata: Salamandridae). Journal of Helminthology, 88, 278-285.

Cagatay IT, Aydin B, Aktop Y, Yilmaz HE. 2022. Molecular and morphologic study of *Clinostomum complanatum* (Digenea, Clinostomidae) in *Garra rufa* (doctor fish) from Southern Turkey. Fresenius Environmental Bulletin, 31, 4791-4800.

Cojocaru C-D. 2009. Fish parasites with significance for ichthyopathology and public health in Romania. 14th EAFP International conference, Diseases of Fish and Shellfish, Prague, September 14-19, 2009.

Çolak HS. 2013. Metazoan parasites of fish species from Lake Sığırcı (Edirne, Turkey). Turkish Journal of Veterinary and Animal Sciences, 37, 200-205.

Dávidová M, Ondračková M, Jurajda P, Gelnar M. 2008. Parasite assemblages of European bitterling (*Rhodeus amarus*), composition and effects of habitat type and host body size. Parasitology Research, 102, 1001-1011.

Djikanović V, Paunović M, Nikolić V, Simonović P, Cakić P. 2011. Parasitofauna of freshwater fishes in the Serbian open waters: a checklist of parasites of freshwater fishes in Serbian open waters. Reviews in Fish Biology and Fisheries, 22, 297-324.

Dorigo L, Saccà E, Beraldo P. 2023. First report for Friuli Venezia Giulia of *Clinostomum complanatum* (Rudolphi, 1814) (Digenea, Clinostomidae) and finding of the species in *Cobitis bilineata* Canestrini, 1886 and *Telestes muticellus* (Bonaparte, 1837). Gortania. Botanica, Zoologia, 45, 65-70.

Dzikowski R, Levy MG, Poore MF, Flowers JR, Paperna I. 2004. *Clinostomum complanatum* and *Clinostomum marginatum* (Rudolphi, 1819) (Digenea: Clinostomidae) are separate species based on differences in ribosomal DNA. Journal of Parasitology, 90, 413-414.

El-Dakhly KM, Hussein NM, El-Nahass E-S. 2018. Occurrence of helminths in the great cormorants, *Phalacrocorax carbo*, in Qena, Egypt. Journal of Advanced Veterinary Research, 8, 6-11.

El-Shahawy IS, El-Seify MO, Metwally AM, Fwaz MM. 2017. Survey on endoparasitic fauna of some commercially important fishes of the River Nile, southern of Egypt (Egypt). Revue de Médecine Vétérinaire, 168, 126-134.

Fedorčák J, Šmiga Ľ, Kutsokon I, Kolarčik V, Koščová L, Oros M, Koščo J. 2019. Parasitic infection of *Cobitis elongatoides* Băcescu & Mayer, 1969 by zoonotic metacercariae *Clinostomum complanatum* (Rudolphi, 1814). Journal of Fish Diseases, 42, 1677-1685.

Gaglio G, Reina V, Gjurcević E, Iaria C, Marino F. 2016. Risk of introduction of *Clinostomum complanatum* (Digenea: Clinostomidae) to Sicily through use of *Cobitis bilineata* (Canestrini, 1865) as live baits. Bulletin of the European Association of Fish Pathologists, 36, 105-110.

Gholami Z, Mobedi I, Esmaeili HR, Kia EB. 2011. Occurrence of *Clinostomum complanatum* in *Aphanius dispar* (Actinoptrygii: Cyprinodontidae) collected from Mehran River, Hormuzgan Province, South of Iran. Asian Pacific Journal of Tropical Biomedicine, 1, 189-192.

Gjurčević E, Kužir S, Valić D, Marino F, Benko V, Kuri K, Matanović K. 2022. Pathogenicity of *Clinostomum complanatum* (Digenea: Clinostomidae) in naturally infected chub (*Squalius cephalus*) and common carp (*Cyprinus carpio*). Veterinarski Arhiv, 92, 339-348.

Goga IC, Codreanu-Bălcescu D. 2011. The trematode *Clinostomum complanatum* (Platyhelminthes: Digenea) identified at the perch from the small reservoirs along the Preajba river. Muzeul Olteniei Craiova. Oltenia. Studii şi comunicări. Ştiinţele Naturii. 27, 115-118.

Gologan I. 2020. Helminth fauna of the European perch - *Perca fluviatilis* (Linnaeus, 1758) from different aquatic biotopes of the Republic of Moldova. Buletinul Academiei de Ştiinţe a Moldovei. Ştiinţele vieţii, 1, 112-118.

Grabda-Kazubska B. 1974. *Clinostomum complanatum* (Rudolphi, 1819) and *Euclinostomum heterostomum* (Rudolphi, 1809) (Trematoda, Clinostomidae), their occurrence and possibility of acclimatization in artificially heated lakes in Poland. Acta Parasitologica Polonica, 22, 285-293.

Juhásová Ľ, Radačovská A, Bazsalovicsová E, Miklisová D, Bindzárová-Gergeľová M, Králová-Hromadová I. 2019. A study of the endohelminths of the European perch *Perca fluviatilis* L. from the central region of the Danube river basin in Slovakia. ZooKeys, 899, 47-58.

Kadlec D, Šimková A, Jarkovský J, Gelnar M. 2003. Parasite communities of freshwater fish under flood conditions. Parasitology Research, 89, 272-283.

Locke SA, Caffara M, Barčák D, Sonko P, Tedesco P, Fioravanti ML, Li W. 2019. A new species of *Clinostomum* Leidy, 1856 in East Asia based on genomic and morphological data. Parasitology Research, 118, 3253-3265.

Maccagno T. 1934. Osservazioni intorno a *Clinostomum complanatum* Rud. Italian Journal of Zoology, 5, 45-60.

Macko J. 1960. Contribution to platyhelminthes fauna of purple heron (*Ardea purpurea* L.). Biologia, 15, 608-612.

Mahdy OA, Abdelsalam M, Abdel-Maogood SZ, Shaalan M, Salem MA. 2021. First genetic confirmation of Clinostomidae metacercariae infection in *Oreochromis niloticus* in Egypt. Aquaculture Research, 53, 199-207.

Maleki L, Heidari H, Ghaderi E, Rostamzadeh J. 2018. Occurrence and description of *Clinostomum complanatum* (Rudolphi, 1819) metacercariae in freshwater fishes from Gheshlagh basin, West of Iran. Iranian Journal of Animal Biosystematics, 14, 91-103.

Menconi V, Manfrin C, Pastorino P, Mugetti D, Cortinovis L, Pizzul E, Pallavicini A, Prearo M. 2020. First report of *Clinostomum complanatum* (Trematoda: Digenea) in European perch (*Perca fluviatilis*) from an Italian subalpine lake: A risk for public health? International Journal of Environmental Research and Public Health, 17, 1389, 1-7.

Monnens M, Halajian A, Littlewood DTJ, Briscoe AG, Artois T, Vanhove MPM. 2023. Can avian flyways reflect dispersal barriers of clinostomid parasites? First evidence from the mitogenome of *Clinostomum complanatum*. Gene, 851, 146952, 1-9.

Oros M, Hanzelová V. 2009. Re-establishment of the fish parasite fauna in the Tisa River system (Slovakia) after a catastrophic pollution event. Parasitology Research, 104, 1497-1506.

Simsek E, Yildirim A, Yilmaz E, Inci A, Duzlu O, Onder Z, Ciloglu A, Yetismis G, Pekmezci GZ. 2018. Occurrence and molecular characterization of *Clinostomum complanatum* (Trematoda: Clinostomidae) in freshwater fishes caught from Turkey. Parasitology Research, 117, 2117-2124.

Soylu E. 2013. Metazoan parasites of perch *Perca fluviatilis* L. from Lake Sığırcı, Ipsala, Turkey. Pakistan Journal of Zoology, 45, 47-52.

Witenberg G. 1944. What is the cause of the parasitic laryngo-pharyngitis in the Near East ("Halzoun")? Acta Medica Orientalia 3, 191-192.

Žitňan R. 1979. Helminths of fishes of Czechoslovak part of the Danube and their epizootological importance. Materials of XXVI. Session of Mixed Commission for Agreement with Fishery in Danube. Budapest, 148-162.
