# Supplementary material for: Population structure of Clinostomum complanatum (Trematoda: Digenea) with new data on haplotype diversity of flukes from Slovakia and Italy
Source: Parasite. 2025 Jan 22;32:3. doi: 10.1051/parasite/2024080 (PMC11752738; doi:10.1051/parasite/2024080)
Supplement: Supplementary file 2 — Supplementary Table 2: Summary of hosts and localities of Clinostomum sinensis (in bold) and Clinostomum sp. originally described as Clinostomum complanatum from Asia. [file parasite-32-3-s2.pdf]

Supplementary Table 2. Summary of hosts and localities of *Clinostomum sinensis* (in bold) and *Clinostomum* sp. originally described as *Clinostomum complanatum* from Asia.

Country (country code) Locality	Host type	Family	Host species	Common name	P (%)	Reference
**South Korea (KR)**						
Cheokgwacheon in Ulju-gun	F	Cyprinidae	*Acheilognathus gracilis*	n. a.	n. a.	Won et al. 2020
Uisong-gun	F	Cyprinidae	*Acheilognathus koreensis*	oily bitterling	47.5	Chung et al. 1995a
Uisong-gun	F	Cyprinidae	*Acheilognathus rhombea*	flat bitterling	88.9	Chung et al. 1995a
Uisong-gun	F	Cyprinidae	*Acheilognathus yamatsutae*	striped bitterling	41.7	Chung et al. 1995a
Uisong-gun	F	Cyprinidae	*Carassius auratus*	goldfish	25.8	Chung et al. 1995a
Nakdong-gang, Sangju-si	F	Cyprinidae	*C. auratus*	goldfish	n. a.	Won et al. 2020
Chatancheon, Yeoncheon-gun	F	Cyprinidae	*Hemibarbus longirostris*	long nose barbel	28.6	Sohn et al. 2015
Uisong-gun	F	Cyprinidae	*Microphysogobio yaluensis*	n. a.	88.9	Chung et al. 1995a
Uisong-gun	F	Cyprinidae	*Pseudorasbora parva*	stone moroko	32.6	Chung et al. 1995a
Taehwagang River	F	Cyprinidae	*P. parva*	stone moroko	6.7	Rim et al. 1996
Uisong-gun	F	Cyprinidae	*Pungtungia herzi*	black striped gudgeon	50.0	Chung et al. 1995a
Chatancheon, Yeoncheon-gun	F	Cyprinidae	*P. herzi*	black striped gudgeon	4.0	Sohn et al. 2015
Uisong-gun	F	Cyprinidae	*Rhodeus uyekii*	bride bitterling	58.3	Chung et al. 1995a
Uisong-gun	F	Cyprinidae	*Squalidus chankaensis*	Khanka gudgeon	50.0	Chung et al. 1995a
Taehwagang River	F	Cyprinidae	*S. chankaensis tsuchigae*	Khanka gudgeon	20.0	Rim et al. 1996
Hyongsangang River	F	Cyprinidae	*S. chankaensis tsuchigae*	Khanka gudgeon	16.7	Rim et al. 1996
Uisong-gun	F	Cyprinidae	*Squalidus gracilis majimae*	Korean slender gudgeon	14.3	Chung et al. 1995a
Yangcheon in Sancheong-gun	F	Cyprinidae	*S. gracilis majimae*	Korean slender gudgeon	n. a.	Won et al. 2020
Kyonganchon Stream	F	Cyprinidae	*Squalidus japonicus coreanus*	short-barbel gudgeon	18.2	Rim et al. 1996
Yangcheon in Sancheong-gun	F	Cyprinidae	*S. japonicus coreanus*	short-barbel gudgeon	n. a.	Won et al. 2020
Wicheon, Gunwi-gun	F	Cyprinidae	*S. japonicus coreanus*	short-barbel gudgeon	n. a.	Won et al. 2020
Uisong-gun	F	Cyprinidae	*Zacco temminckii*	dark chub	1.6	Chung et al. 1995a
Uisong-gun	F	Cobitidae	*Cobitis sinensis*	Siberian spiny loach	38.6	Chung et al. 1995a
Seoul Capital Area	H	–	20-year-old man	–	–	Song et al. 2018
Mokpo, South Jeolla Province	H	–	33-year-old man	–	–	Park et al. 2009
Gwangju, South Jeolla Province	H	–	46-year-old woman	–	–	Lee et al. 2017
Taegu-shi, Daegu Metropolitan City	H	–	56-year-old man	–	–	Chung et al. 1995b
**Japan (JP)**						
**Aichi Prefecture**	**F**	**Cyprinidae**	***C. auratus***	**goldfish**	**n. a.**	**Iwaki et al. 2018**[1]
**Aichi Prefecture**	**F**	**Cyprinidae**	***C. auratus***	**goldfish**	**up to 90**	**Iwaki et al. 2018**[1]
Koyama Pond in Tottori City	F	Cyprinidae	*Cyprinus carpio*	common carp	28.2	Aohagi et al. 1992a
Koyama Pond in Tottori City	F	Cyprinidae	*Carassius cuvieri*	deepbodied crucian carp	43.9	Aohagi et al. 1992a
Tottori City	F	Cyprinidae	*C. cuvieri*	deepbodied crucian carp	20.5	Aohagi et al. 1993
Koyama Pond in Tottori City	F	Cyprinidae	*Carassius gibelio langsdorfi*	silver crucian carp	30.2	Aohagi et al. 1992a
Koyama Pond in Tottori City	F	Cyprinidae	*P. parva*	stone moroko	6.7	Aohagi et al. 1992a
Koyama Pond in Tottori City	F	Cyprinidae	*Rhodeus ocellatus*	rosy bitterling	0.9	Aohagi et al. 1992a
Koyama Pond in Tottori City	F	Cyprinidae	*Rhodeus lanceolatus*	slender bitterling	10.0	Aohagi et al. 1992a
n. a.	F	Lateolabracidae	*Lateolabrax japonicus*	Japanese sea bass	n. a.	Aohagi et al. 1995
n. a.	F	Leuciscidae	*Leuciscus hakonensis*	big-scaled redfin	n. a.	Aohagi et al. 1995

Doai River	F	Adrianichthyidae	*Oryzias sakaizumii*	northern medaka	44.4	Nitta and Ishikawa 2019
Tottori City	B	Ardeidae	*Ardea cinerea*	grey heron	n. a.	Aohagi et al. 1992b
Tottori City	B	Ardeidae	*Egretta garzetta*	little egret	n. a.	Aohagi et al. 1992b
Tottori City	B	Ardeidae	*Egretta intermedia*	median egret	n. a.	Aohagi et al. 1992b
Tottori City	B	Ardeidae	*Nycticorax nycticorax*	night heron	n. a.	Aohagi et al. 1992b
**Aichi Prefecture**	**B**	**Phalacrocoracidae**	***Phalacrocorax carbo***	**great cormorant**	**n. a.**	**Iwaki et al. 2018**[1]
Kashima, Saga	H	–	26-year-old man	–	–	Kitagawa et al. 2003
Tensui-machi	H	–	35-year-old female	–	–	Hirai et al. 1987
Yamaguchi, Ube	H	–	64-year-old man	–	–	Hara et al. 2014
**Taiwan (TW)**						
Dahan River	F	Cyprinidae	*Acrossocheilus paradoxus*	n. a.	31.8	Wang et al. 2017
Dahan River	F	Cyprinidae	*C. auratus*	goldfish	2.1	Wang et al. 2017
Dahan River	F	Cyprinidae	*C. carpio*	common carp	11.1	Wang et al. 2017
Dahan River	F	Cyprinidae	*Hemibarbus labeo*	barbel steed	12.0	Wang et al. 2017
Dahan River	F	Cyprinidae	*Onychostoma barbatula*	n. a.	0.4	Wang et al. 2017
Chu-Pei Culture Station	F	Plecoglossidae	*Plecoglossus alivelis*	ayu sweetfish	n. a.	Lo et al. 1981
Dahan River	F	Xenocyprididae	*Zacco barbata*	n. a.	9.0	Wang et al. 2017
Dahan River	F	Xenocyprididae	*Zacco pachycephalus*	Taiwan Zacco	20.6	Wang et al. 2017
Dahan River	F	Xenocyprididae	*Zacco platypus*	pale chub	16.1	Wang et al. 2017
n. a.	B	Ardeidae	*Gorsakius melanolophus*	Malayan night heron	n. a.	Fischthal and Kuntz 1976
**India (IN)**						
Meerut District	F	Channidae	*Channa punctatus*	spotted snakehead	35.6	Sharma et al. 2011
Aligarh City, Uttar Pradesh State	F	Channidae	*C. punctatus*	spotted snakehead	24.7	Shareef and Abidi 2012
Diamond Harbour, West Bengal	F	Mastacembelidae	*Macrognathus aral*	one-stripe spinyeel	82.5	Bera et al. 2021
Wayanad District, Kerala State	F	Poeciliidae	*Poecilia reticulata*	guppy, rainbow fish	16.3	Prasadan and Sudha Devi 2007
Aligarh City, Uttar Pradesh State	F	Osphronemidae	*Trichogaster fasciatus*	striped gourami	84.3	Khan et al. 2018
**Thailand (TH)**						
Prachinburi Province	F	Osphronemidae	*Trichopodus trichopterus*	blue gourami	n. a.	Tiewchaloern et al. 1999
Prachinburi Province	F	Osphronemidae	*Trichopsis vittatus*	croaking gourami	n. a.	Tiewchaloern et al. 1999
Prachinburi Province	H	–	38-year-old man	–	–	Tiewchaloern et al. 1999
**China (CN)**						
**Hubei Province**	**F**	**Cyprinidae**	***C. auratus***	**goldfish**	**n. a.**	**Chen et al. 2016**[1]
**Meishan Sichuan's Dongpo District**	**F**	**Catostomidae**	***Myxocyprinus asiaticus***	**Chinese sucker**	**64.9**	**Li et al. 2018**[1]

Legend: F, fish; B, bird; H, human; P, prevalence; n. a., data not available; [1] species originally described as *Clinostomum complanatum* but later redescribed as *Clinostomum sinensis* by Locke et al. (2019).
Note: All flukes originally described as *Clinostomum complanatum* need redescriptions and taxonomic revisions.

REFERENCES

Aohagi Y, Shibahara T, Machida N, Yamaga Y, Kagota K. 1992a. *Clinostomum complanatum* (Trematoda: Clinostomidae) in five new fish hosts in Japan. Journal of Wildlife Diseases, 28, 467-469.

Aohagi Y, Shibahara T, Machida N, Yamaga Y, Kagota K, Hayashi T. 1992b. Natural infections of *Clinostomum complanatum* (Trematoda: Clinostomatidae) in wild herons and egrets, Tottori Prefecture, Japan Journal of Wildlife Diseases, 28, 470-471.

Aohagi Y, Shibahara T, Kagota K. 1993. *Clinostomum complanatum* (Trematoda) infection in freshwater fish from fish dealers in Tottori, Japan. Journal of Veterinary Medical Science, 55, 153-154.

Aohagi Y, Shibahara T, Kagota K. 1995. Metacercariae of *Clinostomum complanatum* found from new fish hosts, *Lateolabrax japonicus* and *Leusiscus hakonensis*. Japanese Journal of Parasitology, 44, 340-342.

Bera AK, Das N, Bhattacharya S, Malick RC, Swain HS, Chowdhury H, Sinha A, Manna SK, Sarkar UK, Das BK. 2021. Molecular confirmation of metacercaria of *Clinostomum complanatum* recovered from one-stripe spiny eel *Macrognathus aral*. Aquaculture Research, 52, 4362-4370.

Chen L, Feng Y, Chen H-M, Wang L-X, Feng H-L, Yang X, Mughal M-N, Fang R. 2016. Complete mitochondrial genome analysis of *Clinostomum complanatum* and its comparison with selected digeneans. Parasitology Research, 115, 3249-3256.

Chung D-I, Moon C-H, Kong H-H, Choi D-W, Lim D-K. 1995a. The first human case of *Clinostomum complanatum* (Trematoda: Clinostomidae) infection in Korea. Korean Journal of Parasitology, 33, 219-223.

Chung D-I, Kong H-H., Moon C-H. 1995b. Demonstration of the second intermediate hosts of *Clinostomum complanatum* in Korea. Korean Journal of Parasitology, 33, 305-312.

Fischthal JH, Kuntz R. 1976. Some digenetic trematodes of birds from Taiwan. Proceedings of the Helminthological Society of Washington, 43, 65-79.

Hara H, Miyauchi Y, Tahara S, Yamashita H. 2014. Human laryngitis caused by *Clinostomum complanatum*. Nagoya Journal of Medical Science, 76, 181-185.

Hirai H, Ooiso H, Kifune T, Kiyota T, Sakaguchi Y. 1987. *Clinostomum complanatum* infection in posterior wall of the pharynx of a human. Japanese Journal of Parasitology, 36, 142-144.

Iwaki T, Waki T, Arakawa J, Ogawa K. 2018. The Digenean *Clinostomum complanatum* found from great cormorant *Phalacrocorax carbo* in Japan. Fish Pathology, 53, 132-135.

Khan S, Ahmed S, Serajuddin M, Saifullah MK. 2018. Variation in seasonal prevalence and intensity of progenetic metacercariae of *Clinostomum complanatum* infection in *Trichogaster fasciatus* fish. Beni-Suef University Journal of Basic and Applied Sciences, 7, 310-316.

Kitagawa N, Oda M, Totoki T, Washizaki S, Oda M, Kifune T. 2003. Lidocaine spray used to capture a live *Clinostomum* parasite causing human laryngitis. American Journal of Otolaryngology, 24, 341-343.

Lee GS, Park SW, Kim J, Seo KS, You KW, Chung JH, Moon HC, Hong GY. 2017. A case of endoscopically treated laryngopharyngitis resulting from *Clinostomum complanatum* infection. Korean Journal of Gastroenterology, 69, 177-180.

Li F, Liu X-H, Ge H-L, Xie Ch-Y, Cai R-Y, Hu Z-C, Zhang Y-G, Wang Z-J. 2018. The discovery of *Clinostomum complanatum* metacercariae in farmed Chinese sucker, *Myxocyprinus asiaticus*. Aquaculture, 495, 273-280.

Lo Ch-F, Huber F, Kou G-H, Lo Ch-J. 1981. Studies of *Clinostomum complanatum* (RUD., 1819). Fish Parasitology, 15, 219-227.

Nitta M, Ishikawa T. 2019. Metacercariae of *Clinostomum complanatum* (Platyhelminthes: Trematoda: Clinostomidae), a parasite of the northern medaka, *Oryzias sakaizumii* (Beloniformes: Adrianichthyidae), from Yamagata Prefecture, Japan. Biogeography, 21, 17-21.

Park CW, Kim JS, Joo HS, Kim J. 2009. A human case of *Clinostomum complanatum* infection in Korea. Korean Journal Parasitology, 47, 401-404.

Prasadan PK, Sudha Devi AR. 2007. Yellow grub disease in the ornamental fish, *Poecilia reticulata* (Poecilidae). Journal of Experimental Zoology-India, 10, 405-407.

Rim H-J, Kim K-H, Joo K-H, Kim S-J, Eom KS, Chung M-S. 1996. The infestation states and changing patterns of human infecting metacercariae in freshwater fish in Kyongsang-do and Kyonggi-do, Korea. Korean Journal of Parasitology, 34, 95-105.

Shareef PA, Abidi S. 2012. Incidence and histopathology of encysted progenetic metacercaria of *Clinostomum complanatum* (Digenea: Clinostomidae) in *Channa punctatus* and its development in experimental host. Asian Pacific Journal of Tropical Biomedicine, 2, 421-426.

Sharma B, Rani V, Chaudhary A. 2011. Infection status of *Clinostomum complanatum* (Rudolphi, 1819) metacercaria from *Channa punctatus* of Meerut District. Journal of Applied and Natural Science, 3, 280-283.

Sohn W-M, Na B-K, Cho S-H, Lee S-W, Choi S-B, Seok W-S. 2015. Trematode metacercariae in freshwater fish from water systems of Hantangang and Imjingang in Republic of Korea. Korean Journal of Parasitology, 53, 289-298.

Song HB, Choi MH, Chung EJ. 2018. Human laryngeal infection by *Clinostomum complanatum*. American Journal of Tropical Medicine and Hygiene, 98, 7-8.

Tiewchaloern S, Udomkijdecha S, Suvouttho S, Chnchamsri K, Waikagul J. 1999. *Clinostomum* trematode from human eye. Southeast Asian Journal of Tropical Medicine and Public Health, 30, 382-384.

Wang M-L, Chen H-Y, Shih H-H. 2017. Occurrence and distribution of yellow grub trematodes (*Clinostomum complanatum*) infection in Taiwan. Parasitology Research, 116, 1761-1771.

Won EJ, Lee YJ, Kim M-J, Chai J-Y, Na B-K, Sohn W-M. 2020. Morphological and molecular characteristics of clinostomid metacercariae from Korea and Myanmar. Korean Journal of Parasitology, 58, 635-645.
